# Is there a role of pharmacogenomics in Africa

**DOI:** 10.1017/gheg.2016.4

**Published:** 2016-05-27

**Authors:** A. Matimba, M. Dhoro, C. Dandara

**Affiliations:** 1 Department of Clinical Pharmacology, College of Health Sciences, University of Zimbabwe, Avondale, Harare, Zimbabwe; 2 Pharmacogenetics Research Group, Division of Human Genetics, Department of Pathology and Institute of Infectious Diseases and Molecular Medicine, Faculty of Health Sciences, University of Cape Town, South Africa

**Keywords:** African genome diversity, pharmacogenomics, sub-Saharan Africa

## Abstract

Pharmacogenomics has the potential of transforming clinical research and improving healthcare in sub-Saharan Africa (SSA). The role of African genome diversity and the opportunities for pharmacogenomics research are highlighted and will enable discovery of novel genetic mechanisms and validation of established markers. African genomics and biobank consortia will play an important role in building capacity for pharmacogenomics in SSA.

## Role of Pharmacogenomics in sub-Saharan Africa (SSA)

The growing burden of disease including infectious diseases such as HIV and TB as well as non-communicable diseases such as diabetes, cardiovascular disease and cancer, requires understanding the underlying molecular mechanisms of susceptibility, drug response and resistance towards improving health solutions in SSA [1]. Pharmacogenomics provides an opportunity to identify candidate genes for studying disease progression and outcomes. The possibility of finding these candidates is empowered by the significant impact (effect size) of drug response phenotypes such as adverse drug reactions and treatment outcomes, in contrast to other complex traits such as disease susceptibility and health [2]. The most highlighted impact of genomics is the inter-population variation, particularly the African genomic diversity, which could provide tools for improved understanding of individual variability in disease and pharmacology [3]. Since most studies are carried out on non-African populations (Asians and Caucasians), a smaller portion of the overall human genome's diversity is interrogated and results from replication studies do not always concur. Pharmacogenomics in SSA populations will probe the widest genetic diversity and improve the chances of identifying novel and relevant mechanisms of disease and for targeted therapies to sub-populations.

There are emerging interests in conducting clinical trials in SSA in the future. Currently most medicines are produced in Europe and applied without any further testing in SSA populations and might not be optimally effective or cause severe side effects. Dose adjustments have been recommended for efavirenz due to genetic variations in *CYP2B6* gene, which results in HIV patients in SSA requiring a reduced dose [4, 5]. In future, drug regulations may require population specific studies and pharmacogenomics to evaluate current and new drugs used in SSA.

There is need to investigate and document genetic variation at loci of pharmacogenetic relevance among different SSA populations since this information could be used to inform drug efficacy, safety and recommended dosage [6–8]. Although the trend is to apply whole genome analysis methods, the candidate gene approach provides a powerful tool for validation of pharmacogenetic markers in clinical cohorts in terms of analytical and clinical validity and utility. Candidate gene markers can be selected from existing databases such as the Pharmacogenomics Knowledge Base, which provides a data-sharing resource for the curation of pharmacogenomics knowledge in addition to processing information about potentially relevant markers for clinical pharmacogenetic testing [9, 10]. Over 50 genes have been prioritized as Very Important Pharmacogenes (VIP). To date over 40 gene-drug pairs, including *CYP2D6*–tamoxifen, *TPMT*–azathioprine–6-mercaptopurine, *VKORC1/CYP2C9*–warfarin, *CYP2C19*–clopidogrel, HLA-B*5701–abacavir, HLA-B*5701–flucloxacillin, *SLCO1B1*–statins and *CYP3A5*–tacrolimus have been recommended as actionable for purposes of using genetic information to change prescription of drugs according to the Clinical Pharmacogenomics Implementation Consortium and USA Food and Drug Agency. Multiplex array technology such as the Drug Metabolizing Enzymes and Transporters chip analyses polymorphisms in 225 genes encoding for proteins involved in Absorption, Distribution, Metabolism and Elimination of drugs [11]. However, the challenge is still that most of the panels on the market do not cover some Africa-specific variants. Building awareness about these pharmacogenomics resources may encourage targeted analysis of candidate markers in VIP genes in African populations and clinical cohorts to understand their prevalence and potential impact on drug treatment outcomes. This will promote translational research and development of population-specific guidelines for use of genetic information in optimising drug therapies.

The widespread use of herbal medicinal plants in conjunction with conventional medicines presents further complications and should also be considered in pharmacogenomics research due to herb–drug interactions, which may affect efficacy and toxicity profiles of pharmaceutical drugs [12]. Herbal medicinal plants such as *Moringa oleifera* have shown inhibitory effects of cytochrome P450 enzymes and affect bioavailability of some HIV/AIDS and TB drugs [13, 14]. Microarray-based mRNA expression profiling has shown the effects of herbal medicines on expression of genes in drug response pathways [15]. Dietary phytochemicals may also affect expression and function of drug metabolizing enzymes and transporters. Functional genomics studies are required to understand the molecular mechanisms underlying these interactions and their modification by genetic variation [16].

Networks to conduct genomics research and capacity building such as Human Heredity and Health in Africa (H3Africa) consortium. [17, 18] present opportunities to explore genome-wide approaches for analysis of existing and prospective population-based cohorts. In addition to the 1000 genomes and Hapmap projects, which facilitate curation of human genetic variants, the African Genome Variation Project aims to characterize African genomic diversity [19]. Such work will contribute to large-scale pharmacogenomics studies in Africa towards understanding interethnic variation of drug response.

Biobanking linked to clinical cohorts can play an important role in facilitating pharmacogenomics translation [20]. Although biospecimen collections exist, few biobanks support regional or international exchange, which could promote pharmacogenomics growth in SSA due to regulatory restrictions, limited funding for infrastructure and governance issues. The H3Africa consortium as well as the Biobank and Cohort Building Network and the Bridging Biobanking and Biomedical Research across Europe and Africa, are supporting the establishment of biobank infrastructure and bioinformatics platforms.

Through oragnisations such as the African Pharmacogenomics Research Consortium, which aims to consolidate pharmacogenomics research in SSA, inter-network collaborations could accelerate movement towards application in drug development and clinical use in SSA. Since pharmacogenomic testing holds promise for routine application of personalized medicine, medical education curricula in SSA need to be updated to ensure that health professionals are equipped with a working knowledge of genomic medicine. In addition pharmacogenomics research leadership development, collaboration and exchange, as well as updating ethics and regulatory frameworks, will contribute to sustainable pharmacogenomics programmes for SSA.
